# Supplementary material for: Unmet Need for Oral Corticosteroids Use and Exacerbations of Asthma in Primary Care in Taiwan
Source: Biomedicines. 2022 Dec 14;10(12):3253. doi: 10.3390/biomedicines10123253 (PMC9775049; doi:10.3390/biomedicines10123253)
Supplement: Supplementary file 1 [file biomedicines-10-03253-s001.zip › biomedicines-2026648-supplementary.pdf]

Supplementary Material

Questionnaire Survey for Asthma Exacerbation and Use of Oral Corticosteroid

This questionnaire aims to survey the current status of asthma exacerbations and use of oral corticosteroids in your asthma patients. Please choose one answer that most concurs with your daily practice based on your clinical experience.

PART I. Background of Physicians

1. What kind of healthcare facility do you practice in?
   A. Doctors' office   B. Community hospital

2. What is your medical specialty?
   A. Pediatrics   B. Otorhinolaryngology   C. Family medicine
   D. Internal medicine   E. Pulmonology

3. How many asthma patients do you care for per month?
   A. <50   B. 51–100   C. 101–150
   D. 151–200   E. >200

PART II. Physicians' Perspectives on the Management of Asthma

Section 1. Oral Corticosteroid (OCS) for Acute Exacerbation (AE)

4. What percentage of your asthma patients need OCS to treat AEs per month?
   A. ≤5   B. 6–10   C. 11–20
   D. 21–50   E. >50

5. How many daily doses of OCS (prednisolone or equivalent in mg) do you prescribe to treat an AE?
   A. <20   B. 20–40   C. 41–60
   D. >60

6. How many days of OCS do you prescribe to treat an AE?
   A. <3   B. 3–7   C. 8–14
   D. >14

Section 2. Regular Corticosteroid (OCS) for Asthma Control

7. What percentage of your patients need regular OCS for asthma control per month?
   A. ≤5   B. 6–10   C. 11–20
   D. 21–50   E. >50

8. How many daily doses of OCS (prednisolone or equivalent in mg) do you prescribe as regular doses for asthma control?
   A. ≤5   B. 6–10   C. 11–20
   D. 21–40   E. >40

9. How many months does each treatment course usually take while you prescribe regular OCS for asthma control per year?

A. 0.5–1 B. 1–3 C. 3–6
D. >6

10. What percentage of your asthma patients experience ≥ 2 courses of regular OCS for asthma control per year?
A. ≤5 B. 6–10 C. 11–20
D. 21–50 E. >50

11. Which of the following factors do you think contribute to the regular use of OCS for asthma control (multiple choices allowed)?
A. Disease entity of asthma B. Non-adherence / misuse of inhaler
C. Exposure of allergens / air pollution D. Comorbidity

Section 3: Asthma Control Status

12. What percentage of your asthma patients had ≥ 2 unscheduled visits to the clinic or emergent department due to AEs in the past year?
A. 0 B. 1–5 C. 6–10
D. 11–20 E. >20

13. What percentage of your asthma patients were intubated or admitted to intensive care units due to AEs in the past year?
A. 0 B. 1–3 C. 4–6
D. 7–9 E. ≥10

14. What percentage of your asthma patients used ≥ 3 canisters of short-acting β2 agonists (SABA) in the past year?
A. 0–5 B.6–10 C. 11–20
D. 21 - 50 E. >50

15. What percentage of your asthma patients may benefit from biological agents based on reductions of either regular doses of OCS or frequencies of AE?
A. <1–5 B. 6–10 C. 11–20
D. >20
